# The effect of emergency department procedural sedation on cardiac output: post hoc analysis of a prospective study

**DOI:** 10.1097/MEJ.0000000000000922

**Published:** 2022-06-28

**Authors:** Willemien D. Muller, Ellen van Ieperen, Sophie M. Coffeng, Renate Stolmeijer, Ewoud ter Avest

**Affiliations:** a Department of Emergency Medicine, University Medical Centre Groningen, University of Groningen, Groningen; b Department of Emergency Medicine, Nij Smellinghe Hospital, Drachten, The Netherlands; c Air Ambulance Trust Kent, Surrey and Sussex, Redhill, UK

Procedural sedation and analgesia (PSA) is frequently used in the emergency department (ED) to facilitate painful and unpleasant procedures. PSA is generally achieved by the intravenous (IV) administration of sedatives (propofol and midazolam) or dissociative agents (ketamine), often in combination with short-acting opioids (fentanyl). As administration of sedatives and opioids may result in cardiovascular collapse (hypotension), careful hemodynamic monitoring with ECG [heart rate (HR)] and noninvasive blood pressure measurements is the standard of care [1–3]. Oscillometric blood pressure measurements, however, overestimate blood pressures in hypotension and may be subject to interference (e.g. patient motion or manipulation during procedure) [4]. In addition, measurements are generally performed every few minutes, and as a result, episodes of hypotension may remain undetected initially. Therefore, in this study, we aimed to investigate whether there is a role for noninvasive monitoring of cardiac output as an adjunct to standard hemodynamic monitoring in patients requiring PSA in the ED by investigating the effect of sedative and analgetic drugs administered during PSA on cardiac index (CI).

In a post hoc analysis of a prospective study (registered as NCT03930979), CI, stroke volume (SV) and systemic vascular resistance (SVR) were measured noninvasively in patients who received PSA in the ED of a large teaching hospital in the Netherlands between May 2018 and March 2019. Patients were included when they were more than 18 years of age, were due to receive PSA to facilitate a procedure in the ED and provided informed consent for participation. Patients were excluded if PSA was needed urgently according to the treating physician (no time to obtain informed consent and/or to perform necessary calibration of equipment), if they were pregnant or if they had chronic obstructive pulmonary disease or received oxygen suppletion preinclusion. All sedations were carried out by emergency physicians trained in the conduct of procedural sedation and were performed in compliance with the Dutch national guideline for sedation outside the operating theater [2]. The study was approved by the medical ethical committee of the Medical Center Leeuwarden (protocol number nWMO270).

Measurements of SBP, HR, CI, SV and SVR were performed at baseline and 2, 3 and 5 min after administration of sedative and analgetic drugs using the ClearSight system (Edwards Lifesciences, Irvine, California, USA). The methodology of the ClearSight is based on Nexfin technology of pulsatile unloading of the finger arterial walls using an inflatable finger cuff with a built-in photoelectric plethysmograph that uses pressure to maintain a constant blood volume in the finger. The Nexfin technology used in the ClearSight is validated in multiple studies [5–7]. All investigators received training given by a trainer of Edwards LifeSciences (the manufacturer of Clearsight) in how to operate the device before they were able to include patients for this study.

Primary endpoint of interest was defined as the average change in CI during the first 5 min of PSA. Secondary endpoints were the average change in SV and SVR, and the number of patients exhibiting a clinically relevant (defined as >20%) decrease in CI during the first 5 min of PSA. Based on previous literature, it was estimated that with a mean (SD) CI of 3.5 (0.7) l/min/m^2^, a (clinically relevant) 10% decrease in CI as a result of procedural sedation could be detected with a power of 90% and a type I error rate of 5% when 42 patients or more were included [8]. Changes in hemodynamic variables over time were tested using Friedman’s test with post hoc Wilcoxon signed-rank test and Bonferroni adjustment for multiple comparisons.

During the study period, 91 patients were screened for eligibility. Seventeen patients had one or more exclusion criteria, 14 patients were excluded after screening as no reliable signal for cardiac output monitoring could be obtained and 10 patients had to be excluded after the procedure, as PSA duration was less than 5 min, precluding analysis of the predefined endpoint. In one patient, presedation CI measurements were not recorded on the case report form. Further results refer to the remaining 49 patients. Mean age of the patients was 59 (21) years. American Society of Anesthesiologists (ASA) classification ranged from I to III. None of the patients had preexisting heart failure. Three patients had atrial fibrillation, and 14 patients used vasoactive medication on a daily basis. Most patients received PSA for luxation or fracture reductions. Propofol in combination with fentanyl (*n* = 19) or ketamine (*n* = 21) was most frequently administered for PSA. Median baseline (interquartile range) CI was 3.1 (2.4–3.5) l/min/m^2^.

CI did not drop significantly after medication administration: T = 2 min, 3.0 (2.5–3.7); T = 3 min, 3.0 (2.4–3.6); and T = 5 min, 3.0 (2.3–3.5) l/min/m^2^; *P* = 0.86. Median HR and SV increased transiently during the first 2 minutes of the sedation procedure but returned to baseline values thereafter, whereas median SVR demonstrated a significant and persistent drop after the start of the sedation procedure (Table 1). A more than 20% drop in CI was recorded in five subjects, with the largest drop recorded being 1.00 l/min/m^2^ (32%). However, significant cardiovascular compromise (defined as an SBP < 90 mmHg or HR < 50 bpm) was not registered in any of the patients, and none of the patients received IV fluids and/or vasopressors.

**Table 1:** Effect of emergency department procedural sedation on noninvasively measured haemodynamic variables

Haemodynamic variable	Baseline	T = 2	T = 3	T = 5	*P*
CI (l/min/m^2^)	3.1 (2.4–3.5)	3.0 (2.5–3.7)	3.0 (2.4–3.6)	3.0 (2.3–3.5)	0.86
*N* = 49					
SV (ml)	75 (62–105)	73 (55–99)^a^	75 (59–104)	76 (59–100)	0.02
*N* = 49					
SVR (dyn/s/cm^−5^)	1324 (985–1917)	1212 (838–1580)^a^	1239 (874–1696)^a^	1177 (894–1408)^a^	<0.001
*N* = 48					
SBP (mmHg)	147 (134–164)	132 (117–168)^a^	135 (114–160)^a^	132 (113–154)^a^	<0.001
*N* = 48					
HR (bpm)	74 (64–90)	79 (71–96)^a^	77 (67–94)	75 (67–92)^a^	0.002
*N* = 49					

Data presented as median (IQR).

CI, cardiac index; HR, heart rate; IQR, interquartile range; SV, stroke volume; SVR, systemic vascular resistance.

a Denotes a significant difference after Bonferroni correction in post hoc testing compared to baseline measurements pre-PSA. T2, T3 and T5 represent measurements 2, 3 and 5 min after administration of medication respectively.

These results demonstrate that in the majority of subjects, CI is not significantly affected by drugs (dosages) that are regularly administered for PSA in the ED [9]. The average drop in CI was only small [0.1 l/min/m^2^ (3%)] and most likely the result of a (transient) decrease in SV. The decrease in SV, however, was largely offset by concomitant decrease in afterload (SVR) and an increase in HR, which may have been the result of (residual) pain and awareness associated with the procedure being carried out and/or a direct medication effect]. In our study five patients demonstrated a more than 20% drop in CI after PSA medication was administered. Although this did not result in a clinically significant drop in SBP and/or treatment changes, we cannot exclude that similar drug dosages may result in unwanted hemodynamic effects in patients with a low intravascular volume, patients of older age or patients who suffer from preload-dependent heart conditions. Our findings should be regarded as preliminary and exploratory, due to the limited sample size and the relative homogeneity of the study population, mostly consisting of ASA I and II patients. Confounding factors, such as preload (fasting and IV fluids preprocedure), peripheral vasoconstriction (stress and cold temperatures) or administration of multiple drugs with potentially opposing effects on CO may have influenced our results. Future studies should be carried out to investigate if our findings can be replicated for patients with higher ASA score too, who have a higher potential to drop their CI as a result of PSA and in whom noninvasive monitoring of CI can be more challenging due to dysrhythmia’s (variable beat-to-beat pulse contour analysis) or severe heart failure (profound vasoconstriction) [10].

## Acknowledgements

Purchase of the ClearSight finger cuffs was funded by the MCL research fund. The ClearSight system was made available by Edward Lifesciences. The authors have not declared a specific grant for this research from any other funding agency in the public, commercial or not-for profit sectors. Funding institutions had no role in conception, design or conduct of the study or in collection, management, analysis, interpretation or presentation of the data; or preparation, review or approval of the manuscript.

### Conflicts of interest

There are no conflicts of interest.
